# A tribute to Dr Muriel Fallala

**DOI:** 10.4102/phcfm.v14i1.3656

**Published:** 2022-08-15

**Authors:** Sunanda Ray

**Affiliations:** 1 Department of Medical Education, Faculty of Medicine, University of Botswana, Gaborone, Botswana

Dr Muriel Selma Fallala tragically passed away on 03 November 2021, in Bulawayo, Zimbabwe, as a result of a car accident whilst rushing to deliver a patient. Her death was a big blow to the medical fraternity and robbed the Family Medicine training programme of a crucial facilitator. Dr Wedu Ndebele, Deputy Dean of the Faculty of Medicine, National University of Science and Technology (NUST) said:

She was a very senior colleague, having worked in Bulawayo for the majority of her life. She was among the first specialist family physicians and was instrumental in setting up the training of Family Medicine at NUST.^1^

Born in 1952, Muriel Selma Fallala was the second-eldest child in a family of five. A shy, quiet child, she buried herself in books, attending Methodist Primary School and Luveve Secondary School before leaving to study in Zambia at Nkhumba College and Kenyan College. Joshua Nkomo, the leader of the Zimbabwe African People’s Union (ZAPU) during and after the liberation struggle, assisted young people to receive scholarships abroad to train as professionals in medicine, law, agriculture, engineering and other specialties, allowing them to bring back vital skills and expertise to develop the country after independence. Dr Fallala, a committed supporter of the liberation struggle against the Rhodesian government, was granted a scholarship starting in 1976 to study medicine in the former USSR. She graduated from the Moscow School of Medicine in 1981 and returned home to an independent Zimbabwe.

Her medical career began as a junior doctor at Mpilo Central Hospital in Bulawayo. She then went on to work in obstetrics and gynaecology at Mpilo Central until 1989, then as a medical officer for the Zimbabwe National Family Planning Council Southern Region.

She took up private practice as a general practitioner at Galen House in 1992, where her lasting legacy in Bulawayo was cemented. An epicentre of the community and so much more than just a medical facility, Galen House and its staff cared for entire families and generations. It was common for Dr Fallala’s patients to be people she had brought into the world decades earlier. Nothing can better speak to the consistency of care that was the hallmark of Galen House and Dr Fallala’s lifetime of service.

Dr Fallala was one of four private GPs who self-funded to enrol with the University of Stellenbosch’s Family Medicine MMed programme, intending to set up a similar programme in Zimbabwe. A register of specialist family practitioners was established at the Medical and Dental Practitioners’ Council of Zimbabwe (MDPCZ) in 2015. She graduated in 2014 and was one of the first family practitioners to be entered into that register. Her MMed dissertation was on cervical cancer screening in Bulawayo, which she published in the African Journal of Primary Health Care and Family Medicine.

Dr Fallala was appointed lecturer in 2016 in the Department of Community Medicine at NUST, with responsibility for setting up the MMed Family Medicine programme, as well as continuing her private practice at Gallen House. Her ambition was fulfilled when MMed Family Medicine training was established at the Faculty of Medicine, NUST in Bulawayo and at the University of Zimbabwe College of Health Sciences in Harare, which enrolled their first intakes in 2020. She obtained certificates in Advanced Trauma Life Support and Training of Clinical Trainers, which contributed to her expertise in training the next generation of health professionals.

At the time of her death, Dr Fallala was a member of the Gwanda State University Council, a council member of MDPCZ and a past president of the College of Primary Care Physicians of Zimbabwe (CPCPZ). She served on various boards and committees for the Zimbabwe Medical Association (ZIMA) and the World Organization of Family Doctors (WONCA).

Dr Fallala was declared a provincial liberation hero in recognition of her 40 years of medical service in Zimbabwe.^2^ She is buried at the Lady Stanley Cemetery in Bulawayo.

She is survived by her loving and equally fearless daughter Lily.
